# Mapping Oil and Gas Development Potential in the US Intermountain West and Estimating Impacts to Species

**DOI:** 10.1371/journal.pone.0007400

**Published:** 2009-10-14

**Authors:** Holly E. Copeland, Kevin E. Doherty, David E. Naugle, Amy Pocewicz, Joseph M. Kiesecker

**Affiliations:** 1 The Nature Conservancy, Lander, Wyoming, United States of America; 2 National Audubon Society, Laramie, Wyoming, United States of America; 3 Wildlife Biology Program, University of Montana, Missoula, Montana, United States of America; University of California, Berkeley, United States of America

## Abstract

**Background:**

Many studies have quantified the indirect effect of hydrocarbon-based economies on climate change and biodiversity, concluding that a significant proportion of species will be threatened with extinction. However, few studies have measured the direct effect of new energy production infrastructure on species persistence.

**Methodology/Principal Findings:**

We propose a systematic way to forecast patterns of future energy development and calculate impacts to species using spatially-explicit predictive modeling techniques to estimate oil and gas potential and create development build-out scenarios by seeding the landscape with oil and gas wells based on underlying potential. We illustrate our approach for the greater sage-grouse *(Centrocercus urophasianus)* in the western US and translate the build-out scenarios into estimated impacts on sage-grouse. We project that future oil and gas development will cause a 7–19 percent decline from 2007 sage-grouse lek population counts and impact 3.7 million ha of sagebrush shrublands and 1.1 million ha of grasslands in the study area.

**Conclusions/Significance:**

Maps of where oil and gas development is anticipated in the US Intermountain West can be used by decision-makers intent on minimizing impacts to sage-grouse. This analysis also provides a general framework for using predictive models and build-out scenarios to anticipate impacts to species. These predictive models and build-out scenarios allow tradeoffs to be considered between species conservation and energy development prior to implementation.

## Introduction

Global demand for energy has increased by more than 50 percent in the last half-century, and a similar increase is projected between 2007 and 2030 ([1]). Energy production to meet this demand has resulted in increased habitat fragmentation and increased pressures on biological diversity worldwide ([2], [3], [4]). Such impacts are anticipated to continue, as many renewable and non-renewable energy sources cause habitat fragmentation and disturbance. Fossil fuels will likely remain the largest source of energy with oil, natural gas and coal accounting for 80 percent and non-hydro renewable energy sources (i.e. solar, wind, geothermal) accounting for four percent of global energy supplies in 2030 respectively ([1]). In the United States (US), impacts to species are likely to increase as domestic energy production is encouraged to reduce dependence on foreign energy sources. In the Intermountain West, for example, a doubling of oil and gas development occurred between 1990 and 2007 ([5]). If renewable energy can meet 20 percent of US energy demand, as some predict, the land area required (assuming a turbine density of 5 MW per sq. kilometer) for wind development alone would fragment an estimated 50,000 km^2^ of land ([6]).

Many studies have quantified the indirect effect of fossil fuel usage on climate change and on biodiversity, concluding that a significant proportion of species will be threatened with extinction as a result of increasing temperature ([7], [8]). However, few studies have measured the direct effect of new energy production infrastructure on species ([9]), although many have warned of widespread biodiversity loss resulting from increasing human energy use globally ([10], [11], [12]). While diverse predictive modeling techniques have been applied in recent years to project land cover changes and residential development ([13], [14], [15]) and to predict potential species habitat ([16], [17]), similar techniques have not been applied to model anticipated energy development and impacts to species.

Here we employ land use change build-out scenarios for future energy development demand to quantify future impacts on sage-grouse across six western states. To illustrate this concept, we created a map of oil and gas development potential for portions of 12 states in the Intermountain West and used this map and published projections from federal land management agencies to model future oil and gas build-out scenarios at two levels, as opposed to using expert or stakeholder input to create normative scenarios or “visions” (i.e. [18], [19]). We measured the impacts of the build-out scenarios on populations of greater sage-grouse (*Centrocercus urophasianus*), hereafter referred to as sage-grouse, a species for which energy development impacts have been well-documented ([20], [21], [22]), spatially-comprehensive and long-term data is available, and which is currently being considered for listing under the U.S. Endangered Species Act (ESA) ([23]). When applied as part of the planning process, this approach could be used to highlight areas of biological sensitivity or avoidance areas ([24], [25]) necessary to achieve conservation goals for a species, or to indicate if the proposed development will or will not have significant impacts to a population across its range.

## Methods

### Forecasting oil and gas potential

Because a product of this type was not available, we created a probabilistic classification model of oil and gas resource potential to facilitate landscape-scale analysis. We generated a 1-km^2^ prediction (map) using the nonparametric method ‘Random Forests’, developed to address statistical issues related to over-fit and parameter sensitivity in CART (Classification and Regression Trees) models ([26], [27], [28]). Random Forests uses an iterative Bootstrap with replacement (64% of data per Bootstrap replicate) to construct an ensemble of “weak learners” (CARTs based on a random subsample of data). Prediction is made through a majority vote across the ensemble and not by the familiar rule-set in a traditional CART model. The derivation of a probabilistic output from a classification-based model was introduced in Evans and Cushman ([27]) as an extension of the original Breiman ([26]) algorithm. It has been shown that the Random Forests algorithm can find signals in noisy data, handle large numbers of predictor variables, avoid over fit, and is invariant to parametric assumptions (e.g. spatial autocorrelation, normality) ([26], [27], [29]). Our binary response variable included geospatial data on producing and non-producing oil and gas wells, and a series of topographic, geological and geophysical predictor variables. Recent studies have demonstrated the strength and utility of Random Forests when developing a continuous measure of the probability of occurrence based on a suite of categorical or continuous predictor variables ([27], [29]). Nonparametric classification algorithms such as Random Forests are also ideal for modeling complex, non-linear relationships and avoiding problems of autocorrelation and unknown variable interaction across spatial and temporal scales ([27], [29]).

The six predictor variables used in the model were: geophysical data showing aeromagnetic, isostatic gravity, and Bouguer gravity anomalies, geology, topography and bedrock depth. We chose these variables because they are used by geoscientists to predict where hydrocarbon deposits may occur ([30], [31], [32]). Data on aeromagnetic and gravimetric anomalies depict spatial variations in subsurface rock density and magnetism and indicate features such as buried faults and the depth and location of the sedimentary rocks, both of which can be useful for hydrocarbon resource mapping ([33]). The USGS has conducted low-elevation airborne magnetic surveys since 1946; these data were stitched together by the USGS to form a 1-km^2^ national aeromagnetic map. The USGS generated the gravimetric datasets from thousands of gravity observation stations across the US. The Bouguer gravity anomaly map corrects the gravity station field values for influences on the data such as the Earth's tides and rotation, crustal density, and topography. Isostatic residual anomaly data remove the long-wavelength part of the gravity field to correct for distortions from topographic loads and yield a map more appropriate for near-surface gravity mapping. All geophysical data were downloaded from the USGS at 1-km^2^ resolution for the Coterminous US ([34]). Bedrock geology maps ([35]) show the age, distribution and character of bedrock that lies immediately beneath the soils or surface. We represented bedrock geology using the 1∶5,000,000 scale Generalized Geologic Map of the Coterminous United States downloaded from the USGS national map atlas (http://nationalatlas.gov). Topography data can indicate the location of fold and thrust belts where sedimentary rocks have been deformed by horizontal compression. Once compressed, tightly folded and fractured, reservoir rocks may create pools for oil and gas to form ([36]). We represented topography using 30-m USGS National Elevation Data also downloaded from the national map atlas (http://nationalatlas.gov). The spatial distribution of the rock basement can be approximated using depth to bedrock data from the wells database and indicates, at a coarse-scale, where subsurface valleys and peaks of the basement rock are located. We created a 1-km^2^ cell surface model of depth to bedrock derived from well depth information in the oil and gas wells database using inverse distance weighted interpolation (power = 1; number of points within radius = 12; maximum distance = 5000 meters).

We used data on the producing status of oil and gas wells within a 1-km^2^ grid cell as the binary response variable in our model. We acquired the oil and gas wells database from IHS Incorporated (2007, www.ihsenergy.com) for all states in the study area excluding California and southeast New Mexico, for which we were unable to obtain data. The statewide wells records were merged into a single seamless oil and gas wells file, and non-oil and gas wells (e.g. injection, storage, and unclassified wells) and wells without permits or with drilling in-progress were removed. Using well status code information, wells were attributed as producing (1) and non-producing (0). We created a 1-km^2^ spaced point grid for the study area and each point attributed as producing or non-producing. If both producing and non-producing wells occurred within the 1-km^2^ space around each point, the point was still considered producing. The prediction model was built using this point grid, which was then converted to a raster dataset.

**Figure 1:**
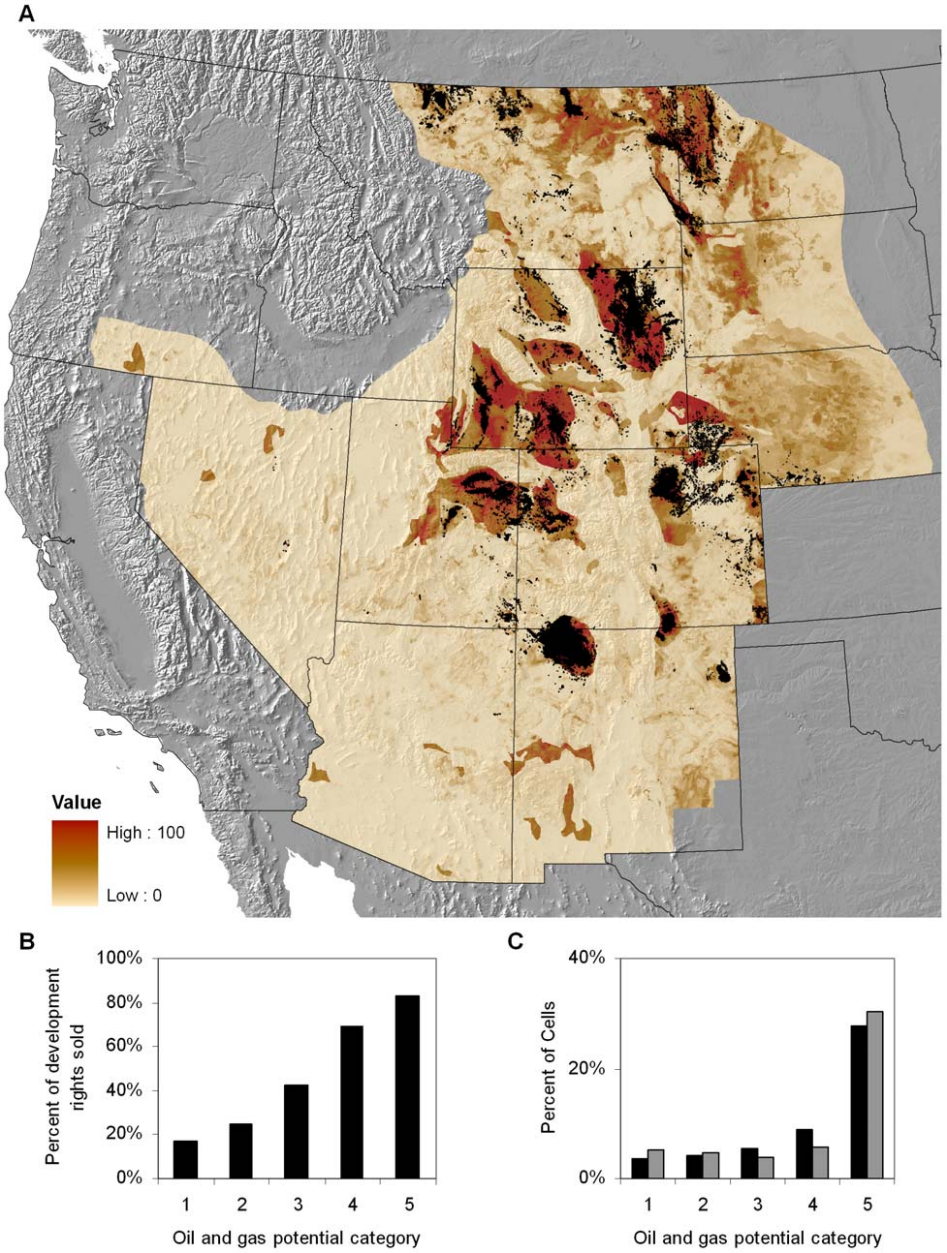
Oil and gas development potential in the US Intermountain West. (A) This map shows the potential for oil and gas development from low to high. Areas in red have the highest potential and tan have the lowest. Black dots show producing (active or inactive) well locations (IHS, Inc.). (B) Percent of federal minerals leased by oil and gas potential category (C) Validation of oil and gas potential model comparing predictions based on pre-1986 data to post-1986 wells drilled by quintile-derived oil and gas potential categories.

We built the oil and gas model using a Random Forests model with 300 bootstrap replicates or classification trees (*k*) and using the entire sample dataset for out-of-bag (OOB) testing with replacement. The number of bootstrap replicates was chosen where OOB error stabilization occurred (between *k* = 200 and *k* = 300 replicates). We avoided software limitations by partitioning the study area into coarse-scale geologic provinces ([37]) and ran the model by province. We applied a balancing algorithm within the Random Forests^TM^ program (Salford Systems, Inc.) to upweight small classes to equal the size of the largest target class and avoid any issues with imbalances between the number of presences and absences with the data parsed by province. Model predictions were first linearly rescaled between 0 to 100, applied to each 1-km^2^ grid cell, and mapped across the Intermountain West as oil and gas development potential where 0 =  low potential and 100 =  high potential (Fig. 1A).

Model validation was performed using OOB testing techniques to produce standard Random Forests model error statistics (area under the ROC curve (AUC) [38], Cohen's kappa [39], OOB error, and class error). The overall average weighted AUC for the two models was 0.889. A model validation statistics summary is presented in Table 1. Accuracy (total number of correct classifications divided by the total number of sample points) varied in the individual models from 79.2 to 86.6% with an overall accuracy of 82.9%. Our cumulative model kappa was 0.61, which shows it to be an acceptable model. As an additional test of our model's prediction ability and to show how well our model would predict without the benefit of more recent data, we built a predictive validation model [40] using well data from 1900–1986 and predictor variables identical to the full model. We tested accuracy of the validation model with well data from 1986–2007. We found that 81 percent of wells producing during 1986–2007 were in areas the validation model predicted for development, which suggests that our model accurately predicts where new wells would be placed up to 20 years into the future (Fig. 1C).

**Table 1:** Accuracy statistics for the oil and gas prediction model.

	AUC	Kappa	OOB Error	Error Class = 0 (non-producing)	Error Class = 1 (producing)
Model 1	0.891	0.60	0.203	27.24	13.40
Model 2	0.886	0.61	0.193	17.77	20.83
Cumulative	0.889	0.61	0.198	22.51	17.12

Our model uses coarse-scale data and thus provides a landscape or regional-scale assessment of oil and gas potential. It cannot predict site-scale potential and model predictions are constrained by the current technology at the time we acquired the wells data (2007) and could be inaccurate if there are significant new advancements in extraction technology.

### Developing build-out scenarios

To predict and locate future oil and gas development, we ran two build-out scenarios—anticipated and unrestrained—by seeding the landscape with oil and gas wells according to the underlying development potential. The US Bureau of Land Management (BLM) is the federal agency responsible for managing mineral development on 283 million acres (including surface and sub-surface mineral estate) of public land in the US. The anticipated scenario was based on 20-year reasonable foreseeable development projections from the BLM's resource management plans (RMPs). Where reasonable foreseeable development projections were unavailable, we calculated resource area estimates by doubling the number of wells permitted from 1996–2007 within a resource area. The unrestrained scenario allowed development in the highest quintile of oil and gas potential (model scores = 75–100). The BLM's estimates have been historically conservative—Colorado's White River Resource Area 1997 RMP predicted 56 wells per year would be drilled, while the actual rate of drilling was three times that since 2004 ([41]). The number of current oil and gas leases across the study area is also indicative that more lands are expected to be developed than RMPs would suggest. Using oil and gas leasing data from the BLM ([42]), we calculated that 81 percent of federal lands with potential for oil and gas development (as defined in this scenario) have already sold their rights (been leased) for oil and gas development (Fig. 1B). Hence, we developed the unrestrained scenario to hedge against these uncertainties.

To place modeled oil and gas wells into the 1-km^2^ cells available for development, we used Community Viz Scenario 360 Allocator Tool (Placeways LLC, Boulder, CO) with the “strict-order allocation” setting, which places wells into the highest probability cells first (using the map of oil and gas potential), then the next desirable, and so on, until all cells have met the specified demand at the specified density. If wells existed in a given cell, the model accounted for those wells in the demand calculation and added new wells until it fulfilled density limitations. The result—the number of wells expected in each cell—is written as an attribute to each 1-km^2^ cell. We excluded lands where oil and gas development is currently prohibited, including National Parks and National Wilderness Areas from the Federal Lands of the United States database ([43]) and “no surface occupancy” (NSO) BLM lands. We mapped NSO lands using a combination of data from the BLM NILS database (Colorado, Montana, North Dakota, South Dakota, Utah) and data from BLM field offices in Wyoming (Wyoming does not contribute this data to the NILS database).

In the anticipated scenario we allocated, per BLM field office, a total 95,867 wells at 16 hectare spacing (32 hectares within coal-bed methane areas of the Powder River Basin, as per current regulations). The unrestrained scenario used the same constraints as the anticipated scenario but placed 260,953 wells in all areas with high oil and gas potential.

### Assessing sage-grouse population and habitat impacts

To demonstrate an application of our predictive oil and gas model, we used the two build-out scenarios to quantify impacts of anticipated and unrestrained development on sage-grouse populations in their eastern range ([44]). Oil and gas development is known to reduce sage-grouse populations at conventional well spacing densities of 16 to 32 hectares ([20], [45], [46]).

To determine whether sage-grouse would be impacted in areas where development occurred, Doherty ([46]) quantified losses of both abundance and occurrence of sage-grouse populations due to oil and gas development by investigating all leks in Wyoming, the largest segment of sage-grouse experiencing oil and gas development impacts in North America. The average responses of leks in Wyoming to different development intensities and amount of time in development compared to control populations experiencing no development were calculated for leks that were active in the last 11 years ([46]: Tables 1 and 2: pp 86–87). We applied the average responses from these tables to all leks throughout Management Zones I and II to predict future losses of sage-grouse to development. We restricted the application of this model to areas within the eastern distribution that were within sage-grouse Management Zone I (Great Plains: includes portions of MT, WY, ND, SD, SA, and AB) and II (Wyoming Basin: includes portions of ID, WY, UT, MT, and CO) ([44], [47]) because these populations are at greatest risk from energy development. We discounted effects of current losses to energy development in calculations of predicted future losses of development by subtracting current losses at specific development intensities to anticipated losses at future development intensities.

Using the build-out scenarios to model impacts to sage-grouse leks as defined above, we predict a 7 percent population decline in the anticipated scenario and 19 percent population decline in the unrestrained scenario compared to 2007 lek population counts. These declines are in addition to the estimated range-wide population declines of 45–80 percent that have already occurred ([47]). The predictions for sage-grouse populations also imply impacts to other sagebrush-dependent species with known sensitivities to oil and gas development such as pronghorn (*Antilocapra americana*) ([48]), mule deer (*Odocoileus hemionus*) ([49]), Brewer's sparrow (*Spizella breweri*), sage sparrow (*Amphispiza belli*) and sage thrasher (*Oreoscoptes montanus*) ([50]).

**Figure 2:**
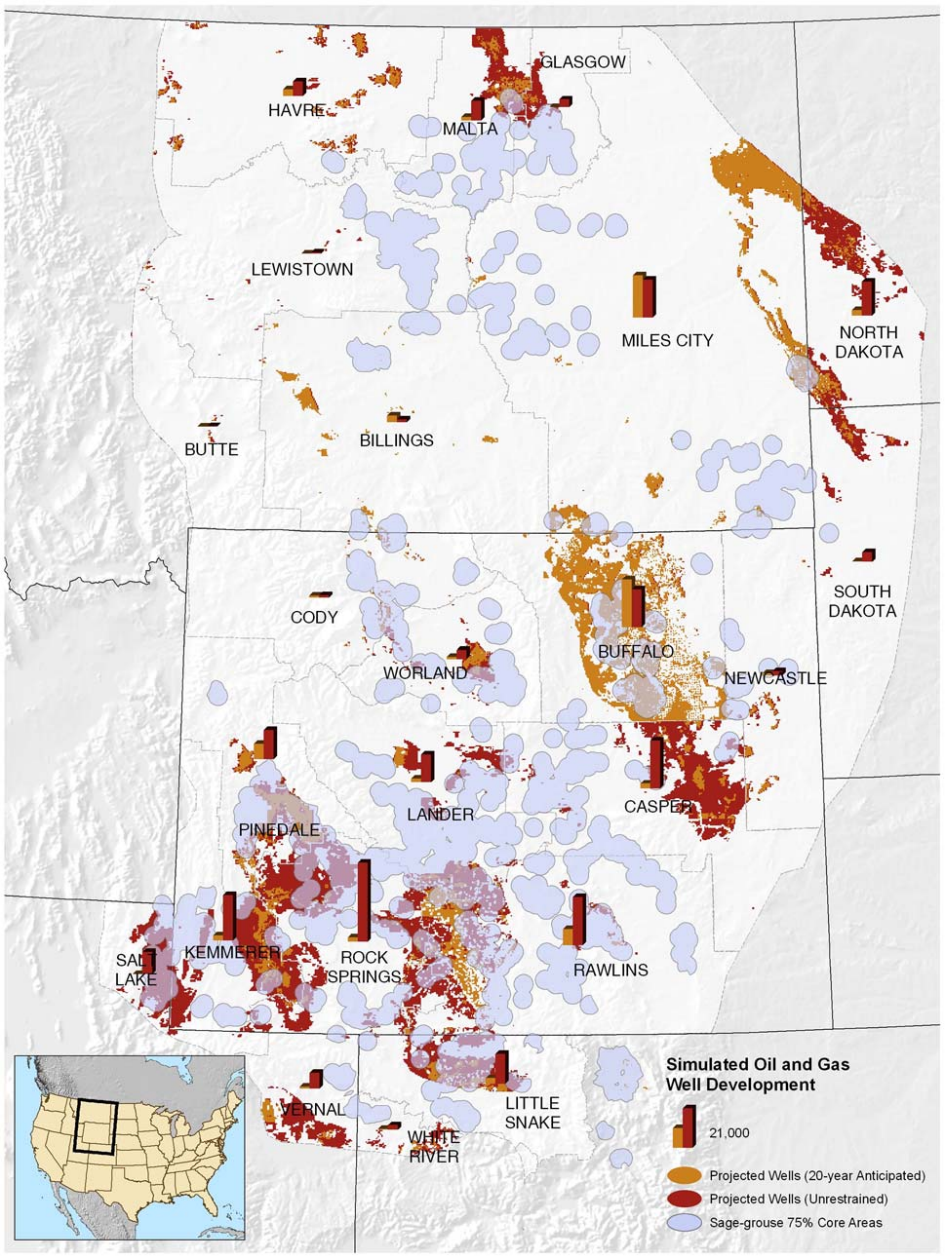
Oil and gas simulation results for the two scenarios. This map illustrates the location and extent of expected development in the two scenarios. Areas in orange depict growth for the anticipated scenario. Areas in red depict growth for the unrestrained scenario. Bar graphs show the quantity of development projected for each scenario. Core areas for sage-grouse are shown to highlight expected areas of future conflict ([46]).

Overall, the anticipated scenario estimates 2.3 million ha (four percent of the study area), an area the size of New Hampshire, will be directly impacted by oil and gas development versus unrestrained scenario impacts of 5.5 million ha or 10 percent of the study area (Fig. 2).We quantified habitat impacts by vegetation type for the unrestrained scenario using Globcover, a regional land cover map for North America ([51]). The habitats predominantly impacted are sagebrush shrublands (3.7 million ha) and grasslands (1.1 million ha), with the remainder a mosaic of hayfields and irrigated croplands.

## Results and Discussion

Our analysis shows that we can expect a 7–19 percent population decline in sage-grouse from future oil and gas development and that the impacts within our study area will be greatest to sagebrush (3.7 million ha) and grassland (1.1 million ha) ecosystems and the species that inhabit them. These results are based on the use of statistical models to forecast future change and the many assumptions inherent to this process. We based our build-out scenarios on projections from the most recent BLM planning documents available at the time and on the oil and gas potential model. BLM estimates are frequently revised from new field discoveries and as technological advances influence resource extraction methods. Forecasted impacts to sage-grouse populations could be revised lower if directional drilling to reduce well pad density at the surface became more commonplace ([52]). Our build-out scenarios are applicable across whole landscapes regardless of land tenure because we assumed that development could occur on any parcel of land, public or private, with the previously noted exceptions. Our estimates provide insights into the trajectory and eventual endpoint of oil and gas development, but the rate and exact location of development will be subject to additional factors not considered such as market demand, the capacity to transport oil or gas to consumers, and federal air and water quality laws (e.g. Clean Air Act, climate change legislation).

**Figure 3:**
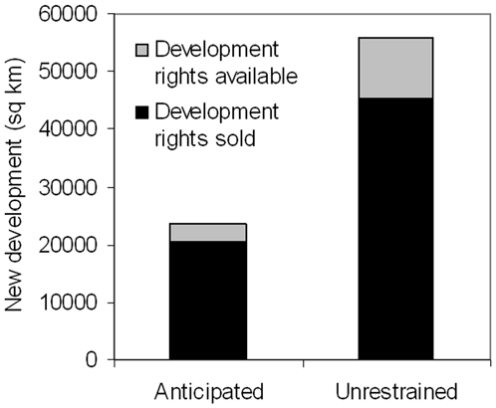
Area of expected development for each scenario. Area (sq km) is divided into development rights sold (black) and development rights available (light gray).

The analysis presented here can be used to inform planners and decision-makers about where oil and gas development is anticipated and potential impacts to sage-grouse. It provides a general framework for analyses using predictive models and build-out scenarios to anticipate impacts to species and the type of information needed for those making decisions about special protections for species, such as ESA listing in the US, and for biodiversity offsets ([53]). The US Fish and Wildlife Service, the agency that oversees ESA listing, faces difficult and complex decisions in determining if current or future risk of species population declines warrants ESA protection. The economic ramifications of listing species are substantial with estimated costs of recovery plans and their implementation reaching into the multi-millions ([54]), if not billions of dollars for wide-ranging species such as sage-grouse. Prevention of listing through thoughtful consideration of threats and possible avoidance or mitigation strategies is likely to be less costly and more effective ([55]). In the case of sage-grouse, 14–19 percent of the study area has high oil and gas development potential but the development rights have not been sold; development in these areas could be avoided by removing these leases for sale or mandating other special protections by government management agencies (Fig. 3). Areas already leased and important for sage-grouse could be considered a priority for lease swaps or buy-backs, where government, non-governmental organizations and other private entities swap land or buy the lease back from the company that bought the development rights. Alternatively, companies could also be encouraged to forfeit their development rights with a perpetual NSO agreement, as part of the negotiation for enhanced access to exploration and development in other areas. Done in the right places, a creative combination of approaches could yield maximum benefit to species.

For many species experiencing population declines, multiple stressors are affecting their populations. The framework we present could be modified to consider not just one type of energy development, in this case oil and gas, but also wind, solar, coal, oil shale and uranium, along with other stressors such as residential development, invasive species, and pathogens. Because many of these stressors do not correlate spatially, this approach would account for cumulative impacts. Models and maps of multiple future threats are needed to fully quantify the future risk to biodiversity.

The case of sage-grouse and oil and gas development in the Intermountain West is a preview of confrontations likely to occur across the globe with profound implications to biodiversity. Incorporating the likelihood of future change into land-use planning can alleviate uncertainty and ultimately make societal adaptation to change more efficient and less costly. Quantifying anticipated future impacts can help to justify proactive protection of places important to biodiversity and to underscore the ecological consequences of failing to do so. We hope to inspire regulatory agencies and land mangers to use technologies available in mapping and modeling to forecast new impacts and for policymakers to use this information to avoid business-as-usual development ([56], [57]), in favor of proactive efforts to predict and avoid impacts in places crucial for species conservation. In the long run, this is likely to be the more ecologically sound, less costly, and more efficient—the more sustainable—course of action.
